# A Meta-Analysis Approach to Defining the Culturable Core of Plant Endophytic Bacterial Communities

**DOI:** 10.1128/aem.02537-21

**Published:** 2022-03-22

**Authors:** Valentina Riva, Francesca Mapelli, Agnese Bagnasco, Alessio Mengoni, Sara Borin

**Affiliations:** a Department of Food, Environmental and Nutritional Sciences (DeFENS), University of Milan, Milan, Italy; b Department of Biology, University of Florence, Florence, Italy; University of Illinois at Urbana-Champaign

**Keywords:** endophytes, plant microbiome, culturomics

## Abstract

Endophytic bacteria are key members of the plant microbiome, which phylogenetic diversity has been widely described through next-generation sequencing technologies in the last decades. On the other side, a synopsis of culturable plant endophytic bacteria is still lacking in the literature. However, culturability is necessary for biotechnology innovations related to sustainable agriculture, such as biofertilizer and biostimulant agents’ development. In this review, 148 scientific papers were analyzed to establish a large data set of cultured endophytic bacteria, reported at the genus level, inhabiting different compartments of wild and farmed plants, sampled around the world from different soil types and isolated using various growth media. To the best of our knowledge, this work provides the first overview of the current repertoire of cultured plant endophytic bacteria. Results indicate the presence of a recurrent set of culturable bacterial genera regardless of factors known to influence the plant bacterial community composition and the growth media used for the bacterial isolation. Moreover, a wide variety of bacterial genera that are currently rarely isolated from the plant endosphere was identified, demonstrating that culturomics can catch previously uncultured bacteria from the plant microbiome, widening the panorama of strains exploitable to support plant holobiont health and production.

## INTRODUCTION

Plants live in association with complex microbial communities, comprising bacterial, archaeal, fungal, and protistic taxa (1, 2). The microbiota and the plant can be collectively defined as holobiont (3). The plant-associated microbiome is so essential for plant health and growth that it is referred to as the second genome of the plant (4, 5). Endophytic bacteria are members of the plant microbiome that spend at least part of their life cycle inside plants (6). They are high-interest targets for sustainable food production and the protection of agri-food systems from biotic stress and adverse environmental conditions. Endophytic bacteria are indeed able to promote plant growth (6), to protect plant against pathogen attack (7–9) and abiotic stresses such as drought, soil salinity and pollution (10).

Culture-independent methods for microbial communities have demonstrated that most environmental microorganisms are recalcitrant to cultivation and constitute the so-called “microbial dark matter.” Non-targeted culture-dependent methods only select for easily culturable and fast-growing taxa, which can represent as little as 1% of the entire diversity (11, 12). In 2019, a provoking brief report fueled the debate about prokaryote culturability across several biomes, including soil but not plant endosphere, arguing that most of the taxa identified in 16S rRNA gene libraries are currently already cultivated (13). The methodology adopted by Martiny (13) was strongly criticized by different authors, which provided contrasting results and demonstrated that a large proportion of bacteria and archaea had not been cultured yet (14). Metataxonomic profiling of plant endophytic bacterial communities are available (2, 15, 16) and metagenomics has been also applied in few studies (1); however. Papik et al. (17) highlighted that endophytes are mainly studied through culture-dependent techniques. Nonetheless, a holistic view of the repertoire of culturable bacterial populations associated with plant endosphere is still lacking in the literature. Genome-driven discoveries have provided new knowledge in the microbial field. Still, most bacterial diversity remains poorly characterized and culturing remains keystone to understand the ecological roles and exploit the biotechnology potential of bacteria (18). For example, culturability of microorganisms whether as isolated strains or in association with their host, in the case of obligate symbionts (e.g., arbuscular mycorrhizal fungi and their associated bacteria [19]), allows the characterization and *in vivo* demonstration of plant growth promotion activity, boosting the development of plant probiotic inoculants in the frame of sustainable agriculture (20, 21). Furthermore, culturable bacteria allow the possibility to establish robust synthetic communities shifting from correlation to causation studies in plant microbiome research (22, 23).

In this perspective, the present study was conceived to collect the information available on the bacteria isolated from plant endosphere, aiming at defining the full extent of their phylogenetic diversity and possibly identifying the presence of a core of culturable endophytic bacteria. A catalogue of cultured endophytes could be a useful tool for researchers working in the field of plant microbiology and specifically for those dedicated to employ bacteria, their metabolisms and products for plant growth promotion and protection finalized to the development of sustainable agriculture practices.

## CREATION OF A DATA SET TO DEFINE THE CURRENT REPERTOIRE OF CULTURED BACTERIAL ENDOPHYTES

Bibliographic research was conducted to establish a large data set (https://doi.org/10.13130/RD_UNIMI/TMTT5S), in December 2020, using Web of Science and Google Scholar databases and combining different keywords. The terms “endophytic bacteria,” “isolation,” and “plant” were searched and crossed with keywords referred to different plant compartment (“seed,” “leaves,” “root,” “shoot”) and specific soil and environmental conditions (e.g., “arid environment,” “polluted soil”). The obtained data set was finally composed by 148 scientific papers (listed in Table S1), that applied well-established surface sterilization procedures, published from 1997 to 2020 (Fig. S1A) and reporting the isolation of endophytic bacteria from plant species distributed among 56 different taxonomic families (Fig. S1B), both *farmed* and *wild* (growing conditions), and at different plant stages. Endophytic bacteria were isolated from different plant compartments: *root*, *shoot*, *leaf*, and *seed.* The studies used plant samples collected around the world from different geographic areas (Fig. S1C) and different climatic zones (identified according to Beck et al. [24]) and, within the data set, we recognized several ecosystems (e.g., wetlands, industrial areas, mountain) and four different soil types (*arid*, *nutrient rich*, *polluted*, and *saline*). The media used for the bacterial isolation were classified according to their “richness” (composition in complex and carbon compounds) in agreement with Oberhardt and coauthors (25): Briefly, considering the concentration cut-off 5 g/L and 15 g/L of complex and carbon compounds, the isolation media were classified as *low*, *medium*, and *high richness media* (Table S2A). Furthermore, media supplemented with metals, salt, and chemicals, besides specific media used for the isolation of nitrogen-fixing bacteria, were categorized as *selective media* (Table S2B). The bacteria isolated in each scientific paper were reported in the established data set at the genus taxonomic level. To the best of our knowledge, this list of bacterial genera provides the first overview of the currently cultured plant endophytic bacteria. The data set is graphically schematized in Fig. S2. The term “record” defines the data collected as each isolation event of a certain endophytic bacterial genus that was retrieved under the different applied isolation conditions in at least one of the 148 scientific articles considered. The records do not account for the abundance of each bacterial genus, while they indicate its presence among the bacterial isolated in the reference scientific literature. The sum of records (i.e., score) of each bacterial genus represents the number of times that it was retrieved in the considered literature (Table S3, Table S4). As shown in Fig. S2, the same genus could be reported by more than one article in the plant endosphere and could be retrieved from different plant species and/or compartments and/or applying different isolation conditions.

## A RECURRENT SET OF CULTURABLE ENDOPHYTIC BACTERIAL TAXA AROSE FROM SCIENTIFIC LITERATURE ANALYSIS

Data derived by high-throughput amplicon and shot-gun metagenome sequencing suggest that plant microbiome is shaped by complex interactions among host, microorganisms, and environment, and that bacterial community composition in plant endosphere changes in response to several biotic and abiotic factors (1, 8). Host plant species, organ and developmental stage, geographical location, soil type, cultivation practice, and fertilization are factors that most significantly influence the structure and diversity of plant endosphere microbiome (26–32). The present work investigates the possible presence of a recurrent set of culturable bacterial genera, independently from the above-mentioned parameters and the conditions adopted in the laboratory for the bacterial isolation from plant endosphere. We hypothesized that a culturable core of plant endophytes can be identified, similarly to the case of human body associated culturable microbiota, composed by numerous bacterial species of which few are frequently isolated under laboratory conditions (33).

**FIG 1:**
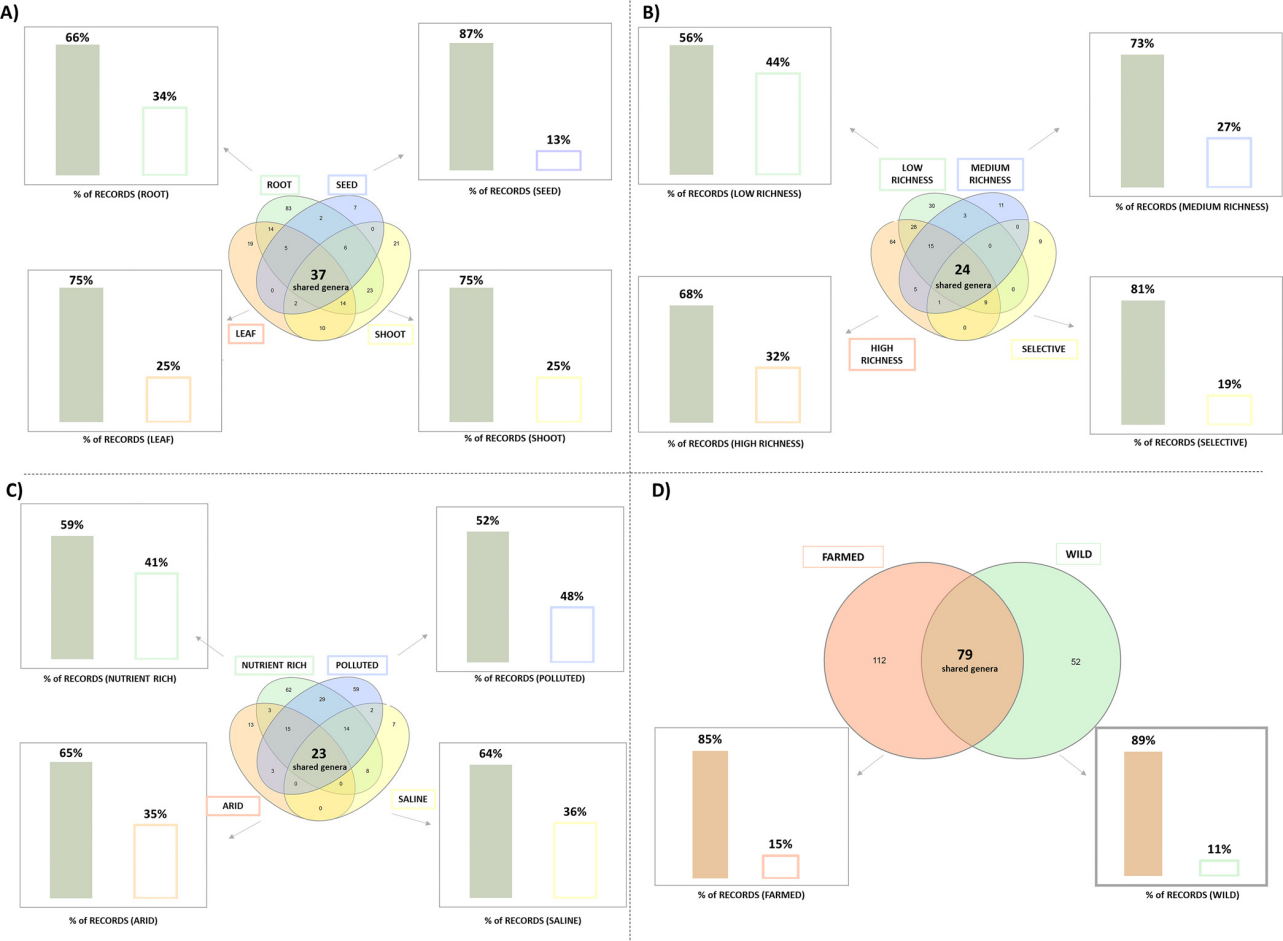
Number of shared bacterial genera and the correspondent record percentage for each comparison. Venn diagrams represent the bacterial genera shared between the categories when comparing (A) plant compartments, (B) isolation media, (C) soil types, and (D) growing conditions. Bar charts indicate, for each comparison, the percentage of records referred to the bacterial genera shared among all the categories (filled bars) or isolated only from some categories (empty bars). Venn diagrams were generated using the open software InteractiVenn at http://www.interactivenn.net/.

**FIG 2:**
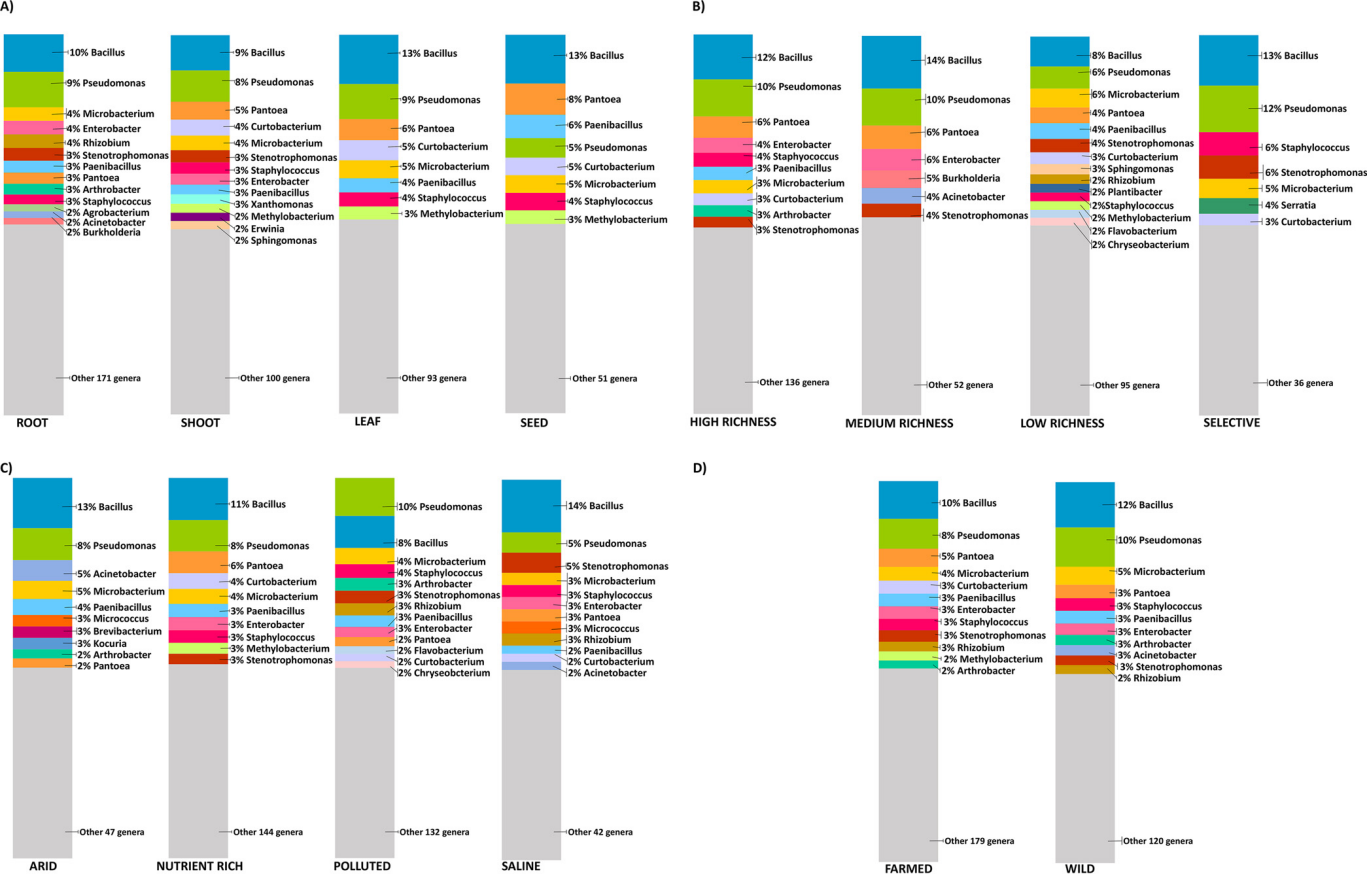
Representation of the genera most represented in the data set. Comparison of the endophytic bacteria isolated (A) from different plant compartments, (B) with different isolation media, (C) from plants grown in different soil types, and (D) from wild and farmed plants. In each panel, the graph shows the phylogenetic affiliation of the most represented bacterial genera up to 50% of records for each category. Each genus is represented with the same color in the different graphs. The gray portion of the bars is the 50% of records that includes the less represented bacterial genera in the categories.

The described bibliographic research generated a catalogue of 243 different bacterial genera isolated from plant endosphere (Table S3). The repertoire of cultured endophytic bacteria was analyzed and compared according to plant compartments, isolation media, soil types, and growing conditions (Table S4A to D). The number of papers for each category can be subjected to variations (Table S4) due, for example, to the relatively recent microbiological investigation as in the case of seed compartment. As shown in Fig. 1, a set of endophytic bacterial genera shared among the different categories was recognized for each comparative analysis performed in this study. Noteworthy, the bacterial genera shared among the categories of each comparison were those predominantly represented, in terms of records. For example, the culturable bacterial genera common to all different plant compartments represented 66% of the total records in *root* category, 87% in *seed* category, and 75% in *leaf* and *shoot* categories (Fig. 1A). Similar results were obtained looking at the bacterial genera common to all isolation media (Fig. 1B), soil types (Fig. 1C), and growing conditions (Fig. 1D), where a remarkable percentage of records (> 50%) represents the bacterial genera shared between all categories. Fig. 2 details the taxonomic genera most frequently reported in the scientific papers included in the data set and representing the 50% of records in each category. *Bacillus* was the bacterial genus most represented (score = 220, Table S3). In all the considered categories, with the exception of *polluted soil* where Pseudomonas was dominant (Fig. 2C), this genus was represented by the highest percentage of records over the total cultured bacterial populations (Fig. 2). Pseudomonas was the second most represented genus in the data set (score = 180, Table S3). These two genera represented cumulatively between the 14% and 25% of the total records in each category (Fig. 2). Bacterial species belonging to *Bacillus* and Pseudomonas genera have been recognized as important plant growth promoters (34, 35) and among those prompting induced-systematic resistance in plants (36, 37).

The bacterial genera most represented in the data set and shared between the analyzed categories belonged to *Gammaproteobacteria* (Pseudomonas, *Pantoea*, *Stenotrophomonas*, and Enterobacter), *Bacilli* (*Bacillus*, Staphylococcus, and *Paenibacillus*) and *Actinobacteria* (*Microbacterium* and *Curtobacterium*) (Fig. 2, Table S4). Similar results were reported in a previous study based on the analysis of 16S rRNA gene sequences longer than 300 bp, derived from both cultivated (56%) and non-cultivated (44%) plant endophytes, with the aim to identify the bacterial genera prevailing in the plant endosphere (6). The coherence between the data presented by Hardoim and coauthors (6) and those illustrated in this study allowed us to exclude a possible bias related to the revision of the taxonomy of certain bacterial families in the past years (for example, those belonging to the order *Enterobacteriales* [38]). Next generation sequencing (NGS) studies generally report data at phylogenetic level higher than the genus, indicating that *Proteobacteria*, *Bacteroidetes*, *Firmicutes*, and *Actinobacteria* phyla include the large majority of bacteria associated to plant tissues (39). Amplicon-based studies are widely used to describe plant microbiome diversity and respond to ecological questions about community assembly, even though they present the inherent biases related to PCR, including the fact that 16S rRNA primer sets are designed on known sequences, with a consequent biased enrichment for known taxa. Moreover, in the endosphere, they can lead to the co-amplification of plastids and mitochondrial DNA from the host (40). On the other hand, shot-gun metagenomics can provide more reliable information about bacterial community composition, but it has been rarely applied to plant endosphere, due to the costs, the interference of host DNA, and the computation effort needed (17, 40). In fact, the metagenomics (and other *omics*) information available refers to a reduced set of plant species and they primarily describe the functional diversity of endophytic bacterial communities (41, 42). Interestingly, when used to describe community composition, metagenomics provided results partially confirming the outcomes of NGS studies: *Proteobacteria* and *Bacteroidetes* were indeed reported as dominant endophytes in sugar beet (8) while *Proteobacteria* and *Firmicutes* were identified as the main components of bacterial communities in the rice endosphere (43). Overall, molecular analyses suggest the presence of a plant core microbiome that lives in close association with the host, regardless the plant species or the environmental conditions, and owning key functional traits of paramount importance for plant fitness (1, 5, 44).

Comparing the composition of culturable bacteria isolated from different plant compartments, a principal coordinates analysis (PCoA) conducted on the top 50% most represented genera suggested a trend of similarity in the composition of culturable endophytes moving from *root* toward the aerial part of the plants, till the *seed* compartment (PCO1 explains 78.9% of total variation, Fig. S3A). In fact, the root endosphere seems to harbor a set of culturable bacterial populations diverse from the other plant organs (Fig. 2A). Here, among the most represented bacterial genera, we retrieved *Rhizobium* and *Agrobacterium*, N-fixing bacteria associated to plant roots; *Burkholderia* which is known as biocontrol agents against root diseases (45); and *Arthrobacter* and Acinetobacter which are among the most abundant bacteria in soil (46, 47), the main source of root endophytic bacteria (48). These genera were found also in other plant compartments, at lower frequencies (Table S4A). The cultivation media is one of the most important factors affecting the phylogenetic diversity of endophytic isolates (17); however, the diversity trend suggested by PCoA of the data set is less clear compared with that observed analyzing plant compartments. The most evident difference in terms of cultured bacteria was the separation of samples on the PCO2, explaining 35.3% of the total variation, in response to the use of *selective media* compared with the others (Fig. S3B). The soil type is considered a primary determinant in the composition of bacterial communities associated to plants (49), and accordingly we observed a separation, along PCO1 explaining 58.9% of total variation, of the bacteria isolated from plants grown in *arid* and *saline* soils and those grown in *nutrient rich* and *polluted* soils (Fig. S3C). Such differences could be due to the fact that both arid and saline soils are poor in nutrients and salinity negatively impact the nutrient assimilation by plants (50), while *polluted* soils, despite the presence of contaminants, can display high resource availability like those classified as *nutrient rich.* The presence of various contaminants (e.g., pesticides, pharmaceuticals, and hydrocarbon compounds) in the polluted soils considered in our data set did not particularly affect the taxonomic composition of the more frequently reported endophytic isolates (Fig. 2C). Likewise, the comparison between the bacterial genera most represented in *farmed* and *wild* plants revealed a similar taxonomic composition (Fig. 2D), even though it is generally acknowledged that plant domestication and breeding have led to changes in the bacterial composition of different crops (51).

## POTENTIAL TO RECOVER PREVIOUSLY UNCULTURED BACTERIA FROM PLANT ENDOSPHERE DOES EXIST

Besides a recurrent set of culturable bacteria, we report the presence of a long tale of culturable bacterial genera that are rarely isolated from the endosphere (Table S4). Fig. 2 showed that, in all the categories, the most represented bacterial genera constituting up to 50% of records were few, while the remaining 50% of records was composed by a very large number of genera. In fact, on average, for each comparison performed in this study, 91% (plant compartments), 88% (isolation media), 86% (soil type), and 93% (plant growth conditions) of bacterial genera were rarely isolated but, collectively, represented 50% of the records. An exhaustive comparison of the different categories focusing on the rarely isolated genera is hampered by the data availability in literature, as previously mentioned. Such comparisons will become feasible by incrementing the studies on culturable endophytic bacteria (52), a direction that will possibly clarify if specific plant species or growth conditions encompass novel bacterial genera endowed by applicative interest, thus representing a hot spot to develop new probiotics for sustainable agriculture. In this sense increasing the phylogenetic diversity of host plants and adopting novel cultivation approaches, which may less target copiotrophic fast-growing bacterial strains, could allow to disclose a still hidden bacterial endosphere diversity.

Notably, a high percentage of the bacterial genera less represented in the data set were singleton (44% of total genera present in the data set, Table S3) as also reported by Hardoim et al. (6) in their catalogue of endophytes. Singleton genera were found in all the categories of the different comparisons with a percentage comprised between 40% and 70% of the whole bacterial genera for each category (Table 1). Interestingly, the percentage of the singleton genera in *saline* category was particularly high (69%) compared with those isolated from plants grown in different soil types (on average 43%) even though such category was represented by lower numbers of scientific papers and host plant families in the analyzed data set (Table S4). Thus, soil type played an influence on the range of rarely cultured endophytes retrieved in the current study and, specifically, saline soils seem to enrich the plant endosphere with a more differentiated cultured bacterial community. Molecular analyses showed that soil salinity plays a selective effect on the overall bacterial community sheltered by the soil (53); however, we cannot exclude that under this condition plants respond widening the panorama of diversity in the endosphere, recruiting those bacteria populations that more effectively can alleviate the stress imposed by salt accumulation (54). Likewise, the percentage of singleton genera isolated using *selective media* was higher (63%) than those isolated on the other medium categories (on average 44%), suggesting that a tailored design of cultivation media has the potential to bring into culture, besides a different composition in the most represented community (Fig. S3B), also a broader range of target endophytic bacteria.

**TABLE 1:** Percentage of singleton bacterial genera present in each category of the analyzed comparisons^*a*^

Category	% of bacterial genera present as singleton
Leaf	57
Root	51
Shoot	44
Seed	56
	
High richness medium	47
Medium richness medium	44
Low richness medium	40
Selective medium	63
	
Arid soil	42
Nutrient rich soil	40
Polluted soil	47
Saline soil	69
	
Farmed plant	38
Wild plant	49

a The table summarizes the relative abundance of singleton bacterial genera over the total cultured genera present in each category.

## CONCLUSIONS AND NEW HORIZONS FOR PLANT MICROBIOME RESEARCH

**FIG 3:**
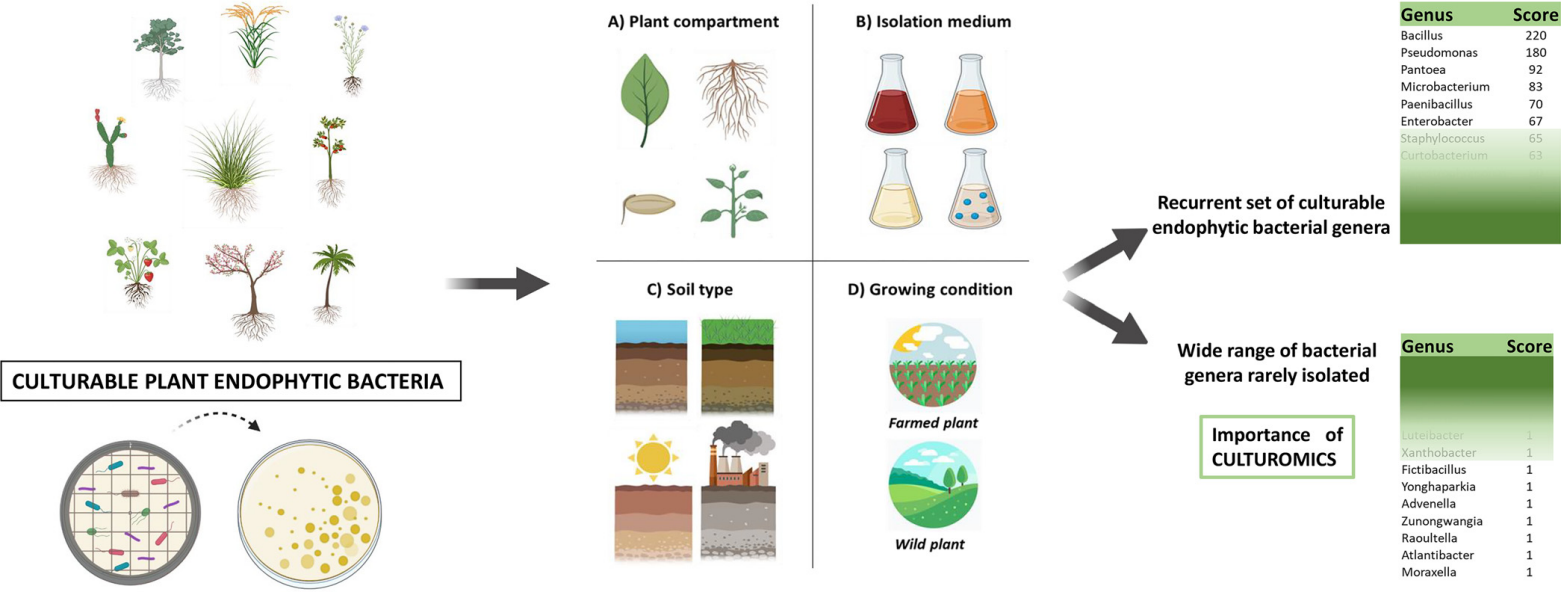
Current situation of culturability in plant endosphere. The scheme indicates the results of this bibliographic study that investigated the taxonomy of culturable endophytes associated to a wide range of plants, and considered different information, i.e., plant compartment, isolation medium, soil type, and plant growing condition. Data elaboration indicates the presence of both a recurrent set of cultured bacterial genera and a wide range of bacterial genera that are rarely isolated under laboratory conditions, revealing the pivotal role of culturomics for the future studies on plant microbiome.

Bacteria dwelling in the plant endosphere are exposed to a dynamic environment due to the plant development and physiological response to fluctuating conditions. In the last years, studies based on 16S rRNA amplicon sequencing extensively described the bacterial community associated to plants, revealing the existence of a core microbiome that is consistently selected by plants, under several conditions. On one hand, it would be worthy to confirm these data by shot-gun metagenomics, on the other we believe that a holistic view of the culturable bacteria able to intimately interact with plants and to colonize their endosphere is still lacking and it would benefit the scientific community striving on the setup of microbial biotechnologies for sustainable agriculture. This study, through an extensive bibliography research, showed that, regardless multiple factors that could influence the diversity of the isolated bacteria, it is possible to recognize a culturable core of plant endophytic bacteria (Fig. 3). Moreover, the adopted meta-analysis approach indicated the presence of a very wide range of bacterial genera that, currently, are rarely isolated from plant endosphere and demonstrated the concrete possibility to bring previously uncultured bacterial species into culture (Fig. 3). The current situation of culturability in plant endosphere seems to recall the scenario of other environments, from marine habitats to human bodies, where culturomics has been successfully applied by microbiologists widening the catalogue of cultured microbes (33, 55). In fact, even though Mycobacterium is still the most frequently cultured genus, culturomics contributed up to 66.2% toward updating the repertoire of isolated human bacteria (33). The culturing strategies developed in the last decades are multiple and the setup of innovative media based on plant materials could be pivotal to enrich and isolate still uncultured bacterial species from the plant microbiome, as shown by the results obtained using plant components alone or as supplements to standard culturing media (56). In the next years, to improve biotechnological exploitation of microorganisms, culturability should not exclusively rely on axenic cultures and, by developing co-cultivation strategies, researchers could jump in a new era of understanding of microbial interactions (57). Moreover, metagenomic data can boost researchers’ ability to pick up novel species from the “microbial dark matter,” as demonstrated by the combination of fluorescence-activated cell sorting with antibodies targeting cell-surface protein to isolate TM7 and SAR1 taxa (58). A comparative study performed on 3,837 high-quality bacterial genomes, derived from plant and other environments, revealed that certain gene clusters are enriched and consistently spread in major phylogenetic groups of plant-associated bacteria, where they identified specific protein domains (59). All in one, omics can both direct the design of ground-breaking culturing effort as well as pave the way to strain engineering aimed at improving root colonization of plant probiotics.
