# Supplementary material for: A Meta-Analysis Approach to Defining the Culturable Core of Plant Endophytic Bacterial Communities
Source: Appl Environ Microbiol. 2022 Mar 22;88(6):e02537-21. doi: 10.1128/aem.02537-21 (PMC8939329; doi:10.1128/aem.02537-21)
Supplement: Supplemental file 5 — Fig. S1 to S3 and legends of Tables S1 to S4. Download aem.02537-21-s0005.pdf, PDF file, 0.6 MB [file aem.02537-21-s0005.pdf]

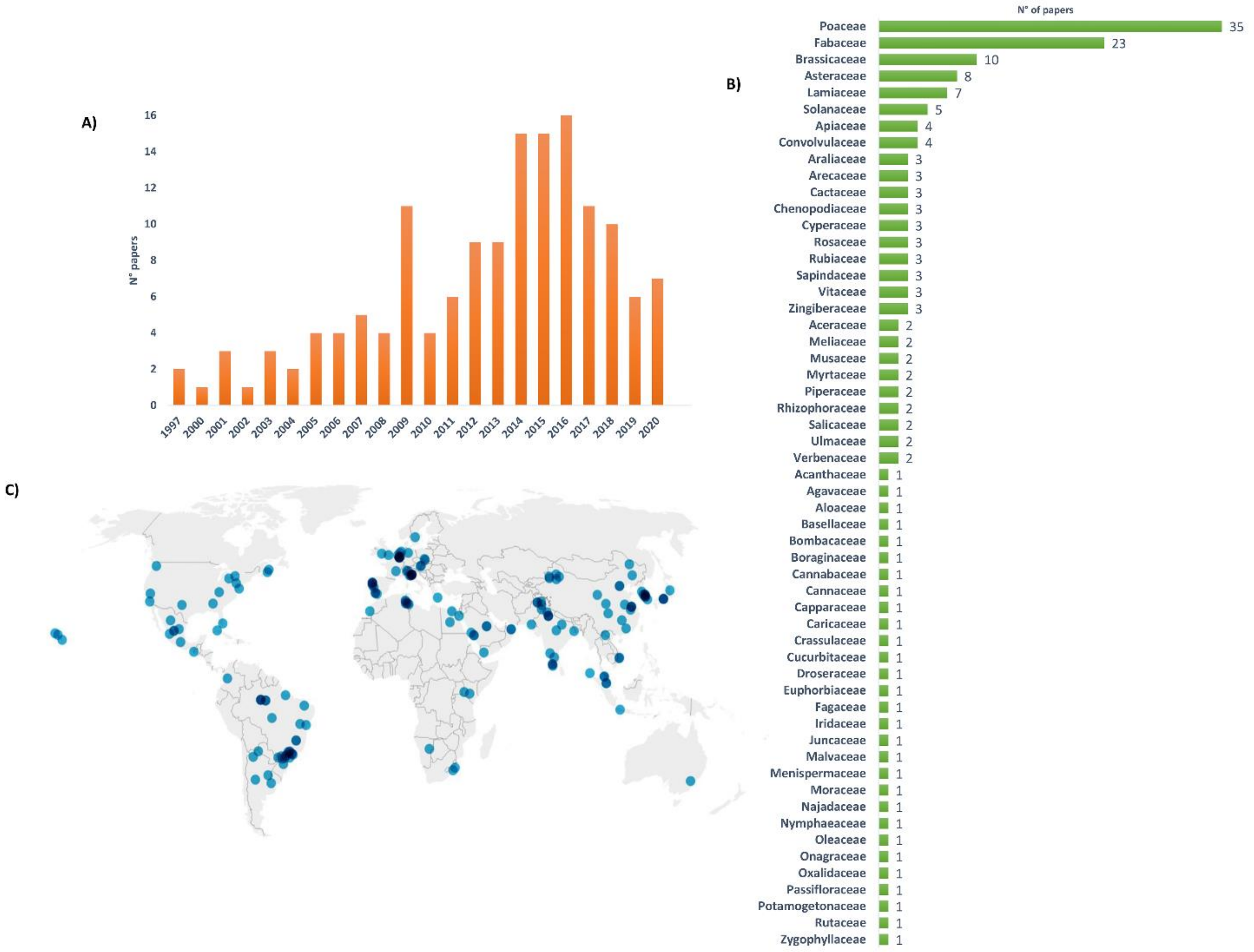

Supplementary Figure 1. General presentation of the dataset. Number of scientific papers reported (A) per year of publication and (B) per plant family and (C) geographic distribution of plants sampled for endophytic bacteria isolation.

References	Plant species	Plant fraction	Medium	...	BACTERIAL GENUS	RECORDS
Paper 1	plant 1	root	mediumA	...	genus 1 (e.g. *Bacillus*)	1
Paper 1	plant 1	root	mediumA	...	genus 2 (e.g. *Pseudomonas*)	1
Paper 1	plant 1	root	mediumA	...	genus 3 (e.g. *Microbacterium*)	1
Paper 1	plant 1	root	mediumB	...	genus 2 (e.g. *Pseudomonas*)	1
Paper 1	plant 1	root	mediumB	...	genus 4 (e.g. *Enterobacter*)	1
Paper 1	plant 2	root	mediumA	...	genus 1 (e.g. *Bacillus*)	1
Paper 1	plant 2	root	mediumA	...	genus 2 (e.g. *Pseudomonas*)	1
Paper 2	plant 3	leaves	mediumC	...	genus 2 (e.g. *Pseudomonas*)	1
Paper 2	plant 3	leaves	mediumC	...	genus 3 (e.g. *Microbacterium*)	1
Paper 2	plant 3	leaves	mediumC	...	genus 5 (e.g. *Pantoea*)	1
Paper 2	plant 3	root	mediumC	...	genus 1 (e.g. *Bacillus*)	1
Paper 2	plant 3	root	mediumC	...	genus 3 (e.g. *Microbacterium*)	1
...	...	...	...	...	...	...

Supplementary Figure 2. Schematic overview of the dataset. The figure exemplifies the categories that can describe each record, showing that the same bacterial genus can be retrieved in different papers and/or under different conditions, representing in this case a new record included in the dataset.

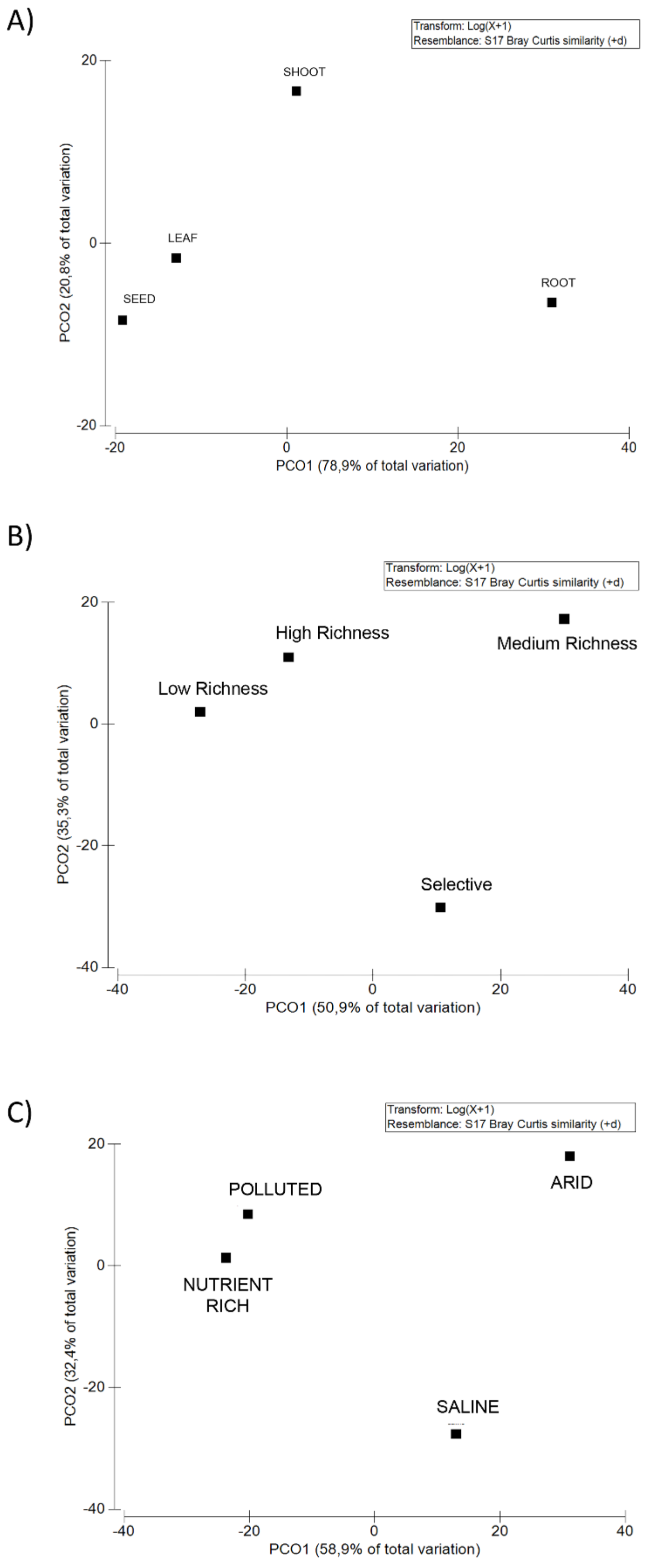

Supplementary Figure 3. Principal Coordinates Analysis (PCoA). PCoA depict the similarity of the samples (i.e., categories) according to their composition in terms of the most represented bacterial genera representing up to 50% of records per each category. The different panels report PCoA results for (A) plant compartments, (B) isolation media and (C) soil types.

Supplementary Table 1. List of the scientific papers included in the dataset. The list includes bibliographic information related to the article (first author, publication year, doi) and the id number of the articles in the dataset for its unambiguous link.

Supplementary Table 2. **Isolation media classification**. (A) List of compounds for each medium. The complex components and carbon compounds are indicated in bold and highlighted in green and their total concentration (g/l) is reported in the last row. Media with a total concentration of complex components and carbon compounds lower than 5 g/l are classified as low richness media (in light orange); media with a total concentration of complex components and carbon compounds comprise between 5 and 15 g/l are considered as medium richness media (in orange); media with a total concentration of complex components and carbon compounds higher than 15 g/l are considered as high richness media (in dark orange). (B) List of media classified as selective.

Supplementary Table 3. Total number of records (score) for each bacterial genus reported in the dataset. Bacterial genera are listed from the most to the less frequently reported ones.

Supplementary Table 4. List of genera and score for each category of the comparisons. The bacterial genera shared among all the categories are indicated in yellow for the comparison between (A) plant compartments, (B) isolation media, (C) soil types and (D) growing conditions. For each category, the numbers of considered scientific papers and plant families are indicated in brackets.
